# Dietary assessment and patient-perspective reasons for poor adherence to diet and exercise post bariatric surgery

**DOI:** 10.1186/s13104-020-05373-y

**Published:** 2020-11-11

**Authors:** Bandar Saad Assakran, Adel Mefleh Widyan, Najla Abdulaziz Alhumaidan, Fadiyah Abdullah Alharbi, Mohammed Abdullah Alhnaya, Abdullatif Abdullah Aljabali, Mohammed Abdulrahman Aleid

**Affiliations:** 1 Bariatric Division, General Surgery Department, King Fahd Specialist Hospital, Alnaziyah, P.O. Box 2290, Buraidah, Qassim 52366 Saudi Arabia; 2 Mathematics Department, College of Science, Qassim University, Buraidah, Qassim Saudi Arabia; 3 Medical Intern, College of Medicine, Qassim University, Buraidah, Qassim Saudi Arabia

**Keywords:** Bariatric surgery, Dietary adherence, Physical activity, Adherence, Barriers

## Abstract

**Objective:**

Obesity prevalence is increasing, and as an outcome, bariatric procedures are on the rise. Previous articles about bariatric surgery disclosed tremendous results. This study aims to assess eating patterns and identify the reasons behind poor adherence to diet and exercise in postbariatric patients.

**Results:**

According to the questionnaire used, the majority (85.5%) of our patients scored “good”, 12% scored “fair”, and only 2% scored “excellent”. None scored “needs improvement”. Fruits had a mean consumption of 1.51 ± 0.79 and vegetables 1.78 ± 0.76. The main reasons for patient nonadherence to healthy eating were low self-discipline (48%), lack of motivation (28%), and availability of healthy food and being too busy to prepare healthy meals, both at 25%. Additionally, 55.9% of the study subjects engaged in physical activity. Lack of time (47%), low self-discipline (38%), and weather (32%) were the primary reasons for not exercising regularly.

## Introduction

A global epidemic has occurred due to poor dietary choices, high caloric diets, low physical activity, or systemic disease [1]. “Obesity” is defined as a body mass index (BMI) of more than or equal to 30 kg/m^2^. It is a major health disease that carries a high risk of many chronic diseases, such as diabetes mellitus, hypertension, and cardiovascular diseases [2]. In Saudi Arabia, the prevalence of obesity is increasing, and it is projected to reach 41% in men and 78% in women by 2022 [3].

Bariatric surgery has shown rising success in the treatment of obesity [4]. It has been the most effective solution for many who have found it ineffective to lose weight through exercise, diet, and other nonsurgical means [4]. Approximately 15,000 bariatric surgeries are performed annually in Saudi Arabia [5]. Antiobesity surgery promotes rapid, significant, and sustainable weight loss along with the remission of obesity-related comorbidities, and it reduces the overall mortality rate by 24.6% [4, 6].

Good compliance with diet and exercise after bariatric surgery is essential to stimulate weight loss, prevent weight gain, avoid malnutrition, and improve quality of life [7]. Sarwer et al. [8] found that postbariatric patients with good dietary adherence were able to lose 28% more of their body weight than those who did not properly adhere. However, a multicenter study of young adults who underwent bariatric surgery revealed that dietary adherence has declined over the years [9]. In one study conducted in sleeve gastrectomy patients, the rate of poor adherence reached 74% by the end of the first year, mainly with low consumption of fruits, vegetables, legumes, and cereals [10]. Similarly, in another study of post-gastric bypass surgery patients, poor dietary choices increased crucially from 11 to 37% in the second year [11].

Bariatric surgery is the most effective treatment in morbidly obese patients, but it is not a panacea, and it should be incorporated with two large domains: “diet and exercise” [12]. As the incidence of obesity is increasing, we aim in this study to assess eating patterns and identify the reasons behind poor adherence to diet and exercise in patients who underwent bariatric surgery.

## Main text

### Methods

After acquiring ethical approval from the hospital administration and local IRB (no. H-04-Q-001), this retrospective cross-sectional study assessed all patients who underwent bariatric surgery from the beginning of 2017 to August 2020, and who had their surgery in a period not less than 6 months at King Fahd Specialist Hospital (KFSH), Qassim, Saudi Arabia. A total of three hundred ninety-nine individuals gave their consent to participate. The inclusion criteria were as follows: patients aged above 18 years old who had bariatric surgery in a period not less than 6 months.

Patients were interviewed via telephone, scheduled for 10–15 min per interview. First, demographic information, including age, sex, occupation, marital status, living status, level of income, level of education, self-reported postoperative weight, and number of obese members in the family, if they had any, were obtained. Patients were asked whether they achieved equal to or more than 30 min per day of physical activity in most of the week in a yes-or-no type of question. Other parameters, including the type of surgery, preoperative height, and weight, were retrieved from the patients’ medical files.

The Healthy Eating Assessment is a reliable short questionnaire adapted from Paxton et al. [13] It measures the overall healthy eating score by identifying eating patterns over the past few weeks. It asks questions on how many times per day the subject eats fruits, vegetables, dairy products, meat, fish, beans, fast food, packaged snacks high in fat, salt, or sugar, chips, crackers, sweet foods (not ones low in fat), sweetened drinks, and the amount of added fat to food. Each question is scored out of 5, and the total score was computed by summing the score of each question, the highest being 50 and the lowest being 10.

When subjects were asked how frequently healthy food was eaten, the higher the frequency the higher the score. Whereas in unhealthy food the lower the frequency the higher the score. For example, if a patient said they consume fruits "once daily" they score a 1, while in sweetened beverages “ones daily” score a 5. A higher score implies that a healthier lifestyle is being practiced. It categorizes patients into four groups: needs improvement (scores 10–19), fair (scores 20–29), good (scores 30–39), and excellent (scores 40–50). A patient who categorizes into either the “needs improvement” or “fair” groups needs to seek help to make better health choices.

Factors behind poor adherence to diet and exercise were studied using a multiple choice question. Choices for not following a healthy diet were as follows: lack of knowledge about healthy food and healthy eating habits, too busy to cook healthy food, lack of motivation, high cost of healthy food, lack of support, poor self-discipline, lack of enjoyment, and low expectations. On the other hand, choices for not following exercise regularly were as follows: lack of time, no access to gym or other training places, lack of enjoyment, socioeconomic status, physical injury, weather, poor self-discipline, and low expectations. In both questions, patients were allowed to share other reasons that were not among the choices previously mentioned.

#### Statistical analysis

Data collected were analyzed using SPSS version 19.0. Quantitative variables are presented as the mean ± SD, and qualitative variables are presented as frequencies and percentages. Student’s t-test and ANOVA were used to compare normally distributed continuous variables, and the Chi-square test (χ^2^) was used to test the association between the categorical variables. A p-value < 0.05 denoted a statistically significant value.

### Results

A total of 399 patients, with an average age of 35.21 ± 10.45, among which 178 (44.6%) were male, and 221 (55.4%) were female. More than half of the study subjects engaged in physical activity. Regarding healthy eating, 341 (85.5%) members of the study sample scored “good”, 50 (12%) scored “fair”, and only 8 (2%) scored “excellent”. None of the samples investigated scored “needs improvement”. Subscales of healthy eating assessment and the participant characteristics are presented in Table 1.

**Table 1:** Participant characteristics and health dietary assessment (n = 399)

Variables	Mean ± SD/ n (%)
Mean age (years) ± SD	35.21 ± 10.45
Sex n (%)	
Male	178 (44.6%)
Female	221 (55.4%)
Occupation n (%)	
Employed	192 (48.1%)
Student	33 (8.3%)
Housewife	83 (20.8%)
Retired	9 (2.3%)
Unemployed	82 (20.6%)
Marital status n (%)	
Single	145 (36.3%)
Married	254 (63.7%)
Living status n (%)	
Living alone	33 (8.3%)
Living with family/other	366 (91.7%)
Income n (%)	
Low	35 (8.8%)
Medium	286 (71.7%)
High	78 (19.5%)
Education level n (%)	
Noneducated	12 (3.0%)
Less than high school	71 (17.8%)
High school and higher education	316 (79.2%)
Mean BMI (kg/m^2^) ± SD	
Preoperative BMI	46.66 ± 12.74
Current BMI	30.91 ± 6.54
Obese member in the family n (%)	
Yes	272 (68.2%)
No	127 (31.8%)
Mean number of obese members in the family	2.42 ± 1.64
Type of surgery n (%)	
Sleeve gastrectomy	390 (97.7%)
Mini gastric bypass	9 (2.3%)
Health dietary assessment total score n (%)	
Excellent	8 (2%)
Good	341 (85.5%)
Fair	50 (12.5)
Needs improvement	0 (0%)
Subscales of healthy eating assessment	
Overall mean of healthy foods in eating habits	3.38 ± 1.005
Fast/fried food or packaged snacks high in fat, salt, or sugar	4.83 ± 0.490
Fruits	1.51 ± 0.786
Vegetables	1.78 ± 0.759
Sweetened beverages	4.28 ± 1.025
Chips or crackers	4.63 ± 0.689
Sweet food or desserts	4.59 ± 0.663
Amount of added fat to food	3.97 ± 0.850
Dairy products	1.66 ± 0.801
Meat/fish/beans	2.80 ± 1.572
Achieve ≥ 30 min per day of physical activity n (%)	
Yes	223 (55.9%)
No	176 (44.1%)

Regarding the variables that were associated with the healthy eating score, only three had a significant association; sex, occupation, and marital status had p-values of 0.027, 0.027, and 0.006, respectively. The mean age of patients was not statistically significant among healthy eating score categories (33.10 ± 11.85 vs. 35.36 ± 10.27 vs. 40.38 ± 8.80, p-value = 0.172) (see Additional file 1:Table S1).

**Fig. 1:**
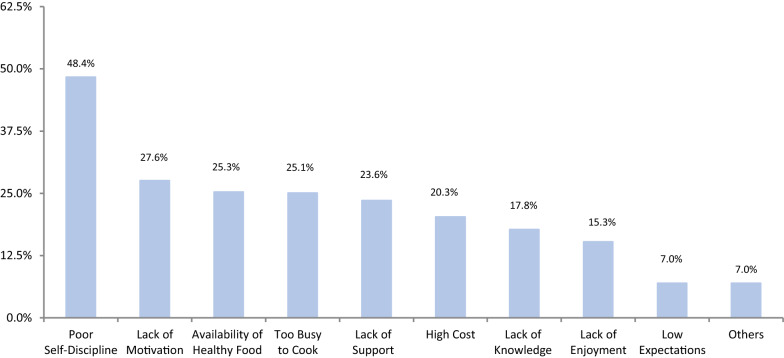
Reasons for not following a healthy diet

**Fig. 2:**
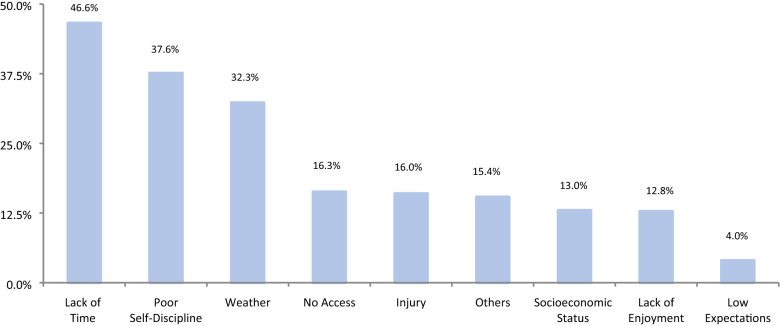
Reasons for not exercising regularly

The main reasons for patient nonadherence to healthy eating were low self-discipline (48%), lack of motivation (28%), and availability of healthy food and being too busy to prepare healthy meals, both at the same percentage (25%) (Fig. 1). On the other hand, lack of time, low self-discipline, and weather were the primary reasons for not exercising regularly, at 47%, 38%, and 32%, respectively (Fig. 2).

### Discussion

By means of structured interviews via phone, we ran a retrospective study on 399 bariatric patients to assess their eating habits and reasons for poor compliance. Bariatric surgery is already known to be very effective. The “forced behavioral changes” in the first few months post-surgery lead to rapid weight loss. However, the results may not last long since gastric and intestinal adaptation are expected to occur 2 years following surgery [14]. Freire et al. [15] revealed some weight gain in the second year, second to the fifth year, and over 5 years to be 14.7%, 69.7%, and 84.8%, respectively. It might be influenced by the reduction in the frequency of dumping syndrome symptoms, resolution of food intolerances, and return to preoperative eating and other lifestyle patterns that initially contributed to weight gain [16].

In the present study, the final healthy eating assessment score was relatively acceptable, as the majority 341 (85.5%) scored “good”, 50 (12%) scored “fair”, and none scored “needs improvement”. General nutritional guidelines post-surgery prioritize protein intake, minimizing high-carbohydrate and high-fat foods, eliminating caloric beverages, and increasing the consumption of fruits and vegetables [17]. Fruits and vegetables provide the body with a wide range of nutrients [18]. In our study, the average intake of fruits and vegetables was “once daily”, 1.51 ± 0.79 and 1.78 ± 0.76, respectively. These averages are lower than recommended. Low consumption of fruits and vegetables has been reported in other extended follow-up studies [18, 19]. Inadequate nutritional intake may lead to hematological, metabolic, and neurological disorders [20–22].

A high percentage (68.2%) of patients in our study had an obese family member, with an average of 2.42 ± 1.64 per family. The frequency of drinking sweetened beverages was “once daily” 4.28 ± 1.02. However, we did not measure the quantity of food/drink consumed; thus, once daily can either be in high quantity, affecting patients’ weight loss, or at minimum to satisfy the appetite. Out of this large sample, eight patients scored “excellent”, which represents only 2%. A comprehensive nutritional education should be delivered for all, both obese and nonobese individuals, supporting those who need to make healthier dietary choices and to improve body health, reaching maximum bariatric treatment efficacy.

Forty-eight percent of participants reported poor self-discipline as their main barrier for not eating healthier. Loss of control over eating is a proxy for binge eating, as postbariatric patients cannot consume large quantities of food in one sitting [17]. Saunders observed that many patients report feelings of loss of control over eating after bariatric surgery and, in some cases, weight gain after several years [23]. Eating disorders necessitate substantial support from a dietitian, a psychologist, and a family member. Nevertheless, patient motivation and willingness to lose weight are important for surgery to be effective [24, 25]. Diet adherence was shown to be successful when patients are highly motivated [26]. Approximately thirty percent of our patients report a lack of motivation. More interestingly, a minority of patients, all of whom were female, stated that they stopped being strict over their lifestyle, not losing more weight in order to maintain their own perspective of body image and prevent having excess skin.

An increase in physical activity after bariatric surgery is beneficial and effective for weight loss, maintaining weight loss, and improving body composition [27, 28]. Being physically active is highly recommended to preserve lean body mass, benefit cardiometabolic risk factors, increase cardiovascular capacity, and aerobic performance [29, 30]. It is advised to exercise at least 150 min per week [31]. A systemic review found that patients who had bariatric surgery while maintaining an exercise routine can lose on average 3.6 kg more than the 1.5 kg observed in a parallel meta-analysis study of nonsurgical weight loss [27, 32]. Patients who exercise can lose on average 3.6 kg more than the 1.5 kg observed in a parallel meta-analysis study of nonsurgical weight loss [27, 32]. In our study, only 55.9% of subjects achieved ≥ 30 min per day of physical activity, which is similar to what has been reported in other studies [9, 15, 18]. On the other hand, 47% of patients stated lack of time as their primary reason for not exercising regularly, followed by low self-discipline and weather, which accounted for 38% and 32%, respectively. In another study, the most commonly endorsed external barriers were time and weather [33].

To conclude, 399 patients were assessed post-bariatric surgery for dietary habits and reasons for poor compliance. The majority scored “good” on the healthy eating assessment, and while none had scored “needs improvement”, and only 2% scored “excellent”. Fruits and vegetables were found to be consumed less than advised. The main reasons for patient nonadherence were most commonly low self-discipline, followed by a lack of motivation. In regard to physical activity, slightly more than half of the patients achieved ≥ 30 min per day of physical activity. Lack of time, low self-discipline, and weather were the primary reasons for not exercising regularly. As the surgical population grows, a global drive should be undertaken to reduce the prevalence of obesity worldwide. We call for more randomized control trials aiming to correct addressed reasons for poor adherence. We recommend the use of new technology to support and motivate patients through video appointments, group therapy, and smartphone applications.

## Limitations

A limitation is that this is a single-center, retrospective cross-sectional, and patient report-based study. Patients may have reported adherence in a socially desirable manner, and this study may also involve recall bias. Assessment of physical activity was limited, as only one question was used. However, the large number of patients being interviewed and the patient-perspective reasons for difficulty adhering provide valuable information for improving the long-term results of bariatric surgery.

## Supplementary information

**Additional file 1: Table S1.** Association between participant characteristics, BMI, and health benefit score.

## Data Availability

The datasets used and/or analyzed during the current study are available from the corresponding author on reasonable request.

## Abbreviations

BMI: Body mass index
KFSH: King Fahd Specialist Hospital
SD: Standard deviation
